# Hsp90, the Concertmaster: Tuning Transcription

**DOI:** 10.3389/fonc.2015.00100

**Published:** 2015-04-28

**Authors:** Nidhi Khurana, Sunanda Bhattacharyya

**Affiliations:** ^1^Department of Biotechnology and Bioinformatics, School of Life Sciences, University of Hyderabad, Hyderabad, India

**Keywords:** Hsp90, transcription, chromatin modifiers, transcription factors, cancer

## Abstract

In the last decade, Hsp90 has emerged as a major regulator of cancer cell growth and proliferation. In cancer cells, it assists in giving maturation to oncogenic proteins including several kinases and transcription factors (TF). Recent studies have shown that apart from its chaperone activity, it also imparts regulation of transcription machinery and thereby alters the cellular physiology. Hsp90 and its co-chaperones modulate transcription at least at three different levels. In the first place, they alter the steady-state levels of certain TFs in response to various physiological cues. Second, they modulate the activity of certain epigenetic modifiers, such as histone deacetylases or DNA methyl transferases, and thereby respond to the change in the environment. Third, they participate in the eviction of histones from the promoter region of certain genes and thereby turn on gene expression. In this review, we discuss the role of Hsp90 in all the three aforementioned mechanisms of transcriptional control, taking examples from various model organisms with a special emphasis on cancer progression.

## Introduction

Considerable progress has been achieved in understanding the cellular role of the major eukaryotic cytoplasmic chaperone, Hsp90. It aids in the folding and stability of numerous classes of proteins (collectively known as clients), under normal as well as stressful conditions. In normal cell, Hsp90 comprises about 2% of the total cellular proteins. However, in stressful condition, its level is increased significantly (up to 10%) with concomitant increase in its activity. Cancer cells experience a variety of stressful conditions like hypoxia, nutrient deprivation, acidosis, high interstitial pressure (1), and consequently, Hsp90 levels are found to be up-regulated in melanoma (2), breast cancer (3), gastric and pancreatic carcinoma (4, 5), ovarian and endometrial carcinoma (6, 7), etc. The increased level of Hsp90 causes chaperoning of the potentially dangerous oncogenic clients that are otherwise metastable. Thereby, Hsp90 impairs the apoptotic signaling in cancer cells. One such candidate is mutant p53, whose stability and intracellular concentration are aided by Hsp90 (8). Experimental findings establish that Hsp90 inhibition by geldanamycin (GA) in rat embryo fibroblast cell lines A1–5 increases the proteolytic turnover of mutant p53 and enhances its nuclear translocation, although it is unable to restore the wild-type transcriptional activity of target genes.

Although Hsp90 is a cytoplasmic chaperone, a small fraction of Hsp90 (about 3% of the total cellular pool) is present in the nucleus. In recent days, the focus has been shifted in understanding the function of Hsp90 in the nucleus. Two decades back, it was first observed that during heat-shock treatment, Hsp90 is specifically localized in the salivary gland of *Drosophila melanogaster* 93D chromosomal locus as well as at the telomere region of *Chironomus thummi* (9). Intriguingly, the fact that its localization to those regions of chromatin was hindered in the presence of transcription inhibitor suggests a role of Hsp90 in transcription during heat-stressed condition. It also assists in the degradation of unfolded or un-required proteins and thereby plays a significant role in maintaining the protein homeostasis in cell. Hsp90 acts as a master regulator of gene expression as it controls the trafficking of steroid hormone receptors to nucleus in a hormone-dependent manner. Recent study shows that Hsp90 and its co-chaperone FKBP51 also promotes hormone-independent nuclear localization of androgen receptor in prostate cancer cells (10) and thereby plays a critical role in progression of prostate cancer. It is observed that in hormone refractory or androgen-independent (AI) prostate cancer cells, a large pool of androgen receptor is translocated into the nucleus even in the absence of androgen and thus leads to the transcriptional activation of target genes resulting in tumor growth (11, 12). The specific inhibitor of Hsp90, 17-allylamino-17-demethoxygeldanamycin (17-AAG), prevents the nuclear localization of androgen receptor in AI tumor at much lower doses than that required to inhibit androgen induced nuclear import of androgen receptors (AR) (13).

In this review, we shall focus on various transcription factors (TF), which interact with Hsp90. Also, we will discuss about the latest understanding on how Hsp90 is involved in regulating chromatin structure and thereby controls gene expression. Although the cellular role of Hsp90 in transcriptional regulation by modulating chromatin dynamics is apparent, its relevance in cancer progression is yet to be appreciated.

## Major Transcription Factors Belong to the Hsp90 Network Society

The role of Hsp90 in transcriptional regulation is foremost attributed to a wide variety of TFs that serve as its clients. One of the ways by which Hsp90 aids in cell survival upon stressed conditions is by regulating the expression profiles of many genes. However, Hsp90 does not do so by binding to DNA as it lacks DNA binding ability. Nevertheless, it chaperones different proteins that act as either activator (like SP1, STAT5) or repressor (for example, Bcl-6) (14, 15) to govern gross transcriptional program (16). TFs serve as tools to regulate different downstream biological processes. Therefore, by providing its services to TFs, Hsp90 is able to regulate multiple pathways simultaneously and hence, plays a vital role in facilitating the progression of many diseases, infections, and cancer (17, 18). When it comes to get hold of processes relevant to cancer, Hsp90 has its branches penetrating into all the six hallmarks of cancer (19). Among the TFs, which serve as Hsp90 clients, NF-κB, STATs, p53, and Bcl-6 (20–26) top the scores owing to the importance of the processes governed by them, which favor malignant transformation. To orchestrate the transcriptional response in a pathway, two or more TFs, which are Hsp90 clients, work together and allow the progression of a pathway dance to their tune. In this light, Hsf-1, which serves as a client of Hsp90 under normal conditions and drives transcriptional programs that are cancer specific, indulges in a positive feedback loop with mutp53 (another Hsp90 client) and endow cancer cells more resistant to proteotoxic stress. The direct interaction between these two proteins in a feed forward loop reinforces tumorigenesis by stabilizing the transcription of *HSPs* that further stabilize EGF, ErbB2, mutp53, and other oncogenes (27). In another scenario, the broad array of clientele of Hsp90 gives it the advantage to regulate the expression of a single protein in different conditions via different TFs. The parallel effect of the TFs upon cellular machinery is witnessed when Hsf-1 and Hif-1 (hypoxia-inducible factor), the clients of Hsp90, regulate the expression of the same protein FoxM1 under different conditions. On one hand, FoxM1 (a key TF for cell cycle progression and a critical molecule for tumor development and progression) is shown to be induced by hypoxia via direct binding of Hif-1 to its promoter sequence, which causes its up-regulation. Induction of FoxM1 leads to promotion of tumor cells proliferation by diminishing nuclear levels of p21 protein and increasing cyclin B1 and cyclin D1 expression (28). On the other hand, FoxM1 is also regulated by Hsf-1 under heat-shock stress conditions and the induction of FoxM1 by Hsf-1 is required for cell cycle progression through regulating the expression of downstream Cdc20, Cdc2, and Cdc25B proteins (29). The importance of Hsp90 in tumor progression is further portrayed by the following study, which reveals that inhibition of Hsp90 leads to the suppression of Lmp1 expression (a major oncogene encoded by Epstein–Barr virus) that plays a crucial role in development of lymphomas. The effect was due to compromised JAK/STAT and NF-κB signaling pathways owing to the repression of STATs and NF-κB TFs upon Hsp90 inhibition (30).

Hsp90 has long been known to regulate transcription when it comes to steroid hormone signaling and was studied extensively (31). The relevance of steroid hormone receptors in cancer is very well reflected by estrogen and progesterone receptors in breast cancer, and by AR in prostate cancer (32, 33). However, Hsp90 does not fail to add one more layer of regulatory step in stabilizing AR by involving breast carcinoma amplified sequence 2 (Bcas2). Bcas2 is a transcriptional cofactor of estrogen receptor (ER), which is involved in breast cancer malignant progression and also overexpresses in prostate cancer. A recent study reports that Bcas2 interacts with Hsp90 to bring about AR stabilization in a p53-independent manner (34). Hsp90 not only stabilizes its clients but also helps them to localize in the right compartment in the cell where their function is required. This aspect was explicitly shown in a study with a *bona fide* client TF, AF9, which is vital for hematopoiesis. It is also called master regulator of *HOX* gene expression. It is observed that it depends on Hsp90 for proper sub-cellular localization (35). Nevertheless, another study exemplifies the role of Hsp90 in deciding the fate of cell death whether it would be necrosis or apoptosis. The inhibition of Hsp90 dictates the inhibition of Atf3 (a TF that regulates gene expression in response to oncogenic stresses) expression, which regulates the switch from necrosis to apoptosis (36). In these ways, the parameters of *HSP90* regulation are extended to the normal cellular processes as well.

**Figure 1:**
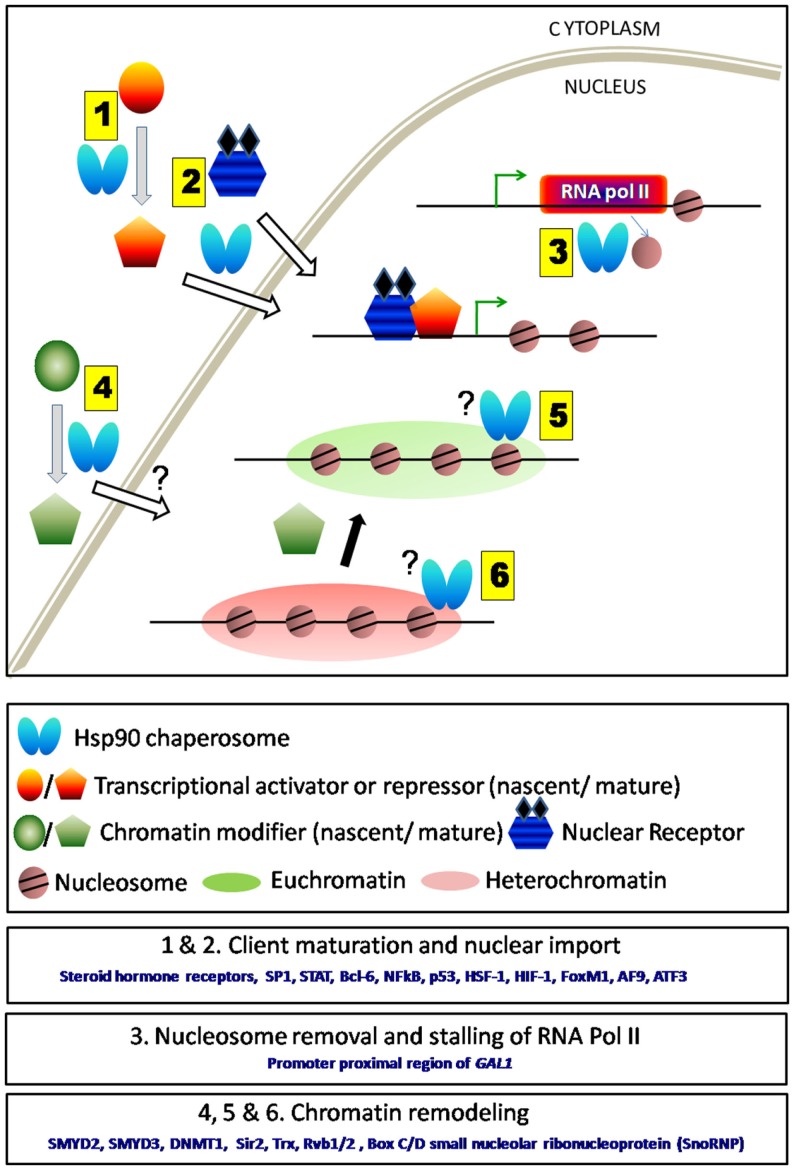
**Schematic representation of direct and in-direct roles of Hsp90 in transcription**. (1) Hsp90 and its co-chaperones aid in the folding of various transcription factors either activators or repressors. (2) It also assists their nuclear transport. (3) Hsp90 being present at the TSS regulates transcription either by nucleosome removal or by stalling RNA polymerase II. Removal of Hsp90 complex thereby allows the movement of RNA polymerase II and initiates gene transcription. (4) Hsp90 aids in the maturation and enhances the activity of several chromatin modifiers. However, it is not clear whether it assists their entry into the nucleus. (5 and 6) Chromatin modifiers upon maturation by Hsp90 are responsible for maintaining euchromatin or heterochromatin states under various conditions. Hsp90 remains associated with some chromatin modifiers (e.g., Trx) near actively transcribing gene.

The versatile nature of Hsp90 does not restrict it to stay “in-house” rather reflects its ability to tune the transcriptional program being “outdoor.” This particular molecular chaperone now is reported to be secreted out in extracellular “reactive” stroma by tumor cells and also under other stressed conditions. This secreted form of Hsp90, addressed as eHsp90, sustains cancer cell motility, invasion, and metastatic spread (37). The extent of secretion of eHsp90 is more in aggressive tumors as it is reported in prostate cancer (38). A recent study suggests eHsp90 as a potent initiator of stromal inflammatory response, which is executed by transcriptional modulation of NF-κB and STAT3, the master regulators of inflammatory pathway (39). Thus, Hsp90 creates a hub of regulatory network where not only the client TFs lead to required alteration in the progression of pathway but also cross-talks among client proteins dictate the downstream effectors for better response to stress stimuli. Thus, Hsp90 regulates the activity of several key transcriptions factors involved in cancer progression via two different mechanisms: in the first place, by regulating the cellular abundance of these factors and second, by regulating their intracellular transports (Figure 1, 1 and 2).

## Communication between Hsp90 and Chromatin Remodeling Factors

So far, we have discussed the role of Hsp90 in transcriptional regulation by directly modulating the activity of TFs. Now, we will discuss how Hsp90 alters the epigenetic marks on chromatin and thereby modulates transcription of several genes that might include proto-oncogenes. Abnormal methylation marks on DNA, altered histone modifications, or RNA-mediated silencing could potentially result in inappropriate gene expression. Any of these epigenetic abnormalities might cause development of cancer. There are increasing amounts of evidence that suggest mutual cross-talks between Hsp90 and several chromatin modifiers (Figure 1, 4). Hsp90α is shown to interact and enhance the activity of (H3–K4) histone methyltransferase (HMTase) SMYD3 whose over-expression is essential for the growth of colorectal-, liver-, and breast cancers (40, 41). Hsp90 induces a conformational change of SMYD3 upon binding to its N-terminal domain, which is essential for the regulation of its cognate HMTase activity (42). It is also reported that the tetratricopeptide repeat (TPR) present at the C-terminal domain of SMYD3 is involved in the physical interaction with MEEVD regions of Hsp90. This interaction is proved to be essential for the chromatin localization and enhancement of HMTase activity of SMYD3 (43). It has been speculated that disruption of the interaction between Hsp90α and SMYD3 might be responsible for inactivation of WNT gene transcription (44). Recent findings show that increased ATPase activity of Hsp90 by Aha1 results in enhanced expression of WNT target genes in colon cancer in a p53-dependent manner (45). It has also been observed that functional inactivation of Hsp90 or post-translational modification of Hsp90 leads to the dysfunction of several chromatin remodelers, which eventually cause alteration of chromatin state associated with many oncogenes and tumor suppressor genes. The “maintenance” methyltransferase DNMT1 is stabilized by Hsp90. Elevated level of DNMT1 is observed in MCF-7 breast cancer cells (46). DNMT1 along with HDAC1 and (H3–K9) HMTase remain associated with the ER-α promoter, causing hypermethylation of 5′ C_p_G islands and thereby causes silencing of ER-α expression in breast cancer cells (47, 48). Studies with HDAC1 inhibitors reveal that post-translational modification (hyperacetylation) of Hsp90 destabilizes its interaction with DNMT1 and promotes ubiquitin-dependent degradation of DNMT1 (49).

In lower eukaryotes like *Saccharomyces cerevisiae*, genome-wide two-hybrid interaction study revealed that Hsp90 may influence global gene expression through interactions with histone deacetylases. Strong association between Hsp90^E33A^ and Sir2 (Type III histone deacetylase) as well as Sap30 (a component of Rpd3L histone deacetylase complex) has been observed (50). Recent studies have established that Hsp90 is required for the stability and functional activity of Sir2. In Hsp90 loss of function mutant, the endogenous level of Sir2 reduces considerably and it results in de-repression of silencing at telomeres and at the mating loci *HML*α and *HMRa* (Figure 1, 6). The temperature-sensitive mutant of Hsp90 behaves similarly as Δ*sir2* mutant resulting in sterile yeast (51). On the other hand, Hsp90 over-expression, which is a natural outcome of heat-stressed condition, drives downregulation of *SIR2* at the transcription level (52). Such reduced abundance of *SIR2* transcript is maintained through several generations before it gradually returns to its normal level. Hence, the level and activity of the chromatin modifier Sir2 are modulated by two independent pathways both controlled by Hsp90. In addition to the regulation of histone deacetylase activity, Hsp90 chaperosome also amends the activity of other types of chromatin modifiers. Two co-chaperones of Hsp90; Tah1 (human ortholog RPAP3) and Pih1 (also known as NOP17 and Pih1D1) are found to interact with Rvb1/2 (53), which are the essential components of INO80 (42, 54); SWR-C chromatin remodeling complex (55–57); and histone acetyl transferase TIP60 complex (58). In *Drosophila melanogaster*, it has been reported that Hsp90 interacts with Trithorax G, which is an important chromatin modifier complex that controls *Drosophila* development. Inhibition of Hsp90 function by radicicol causes depletion of intracellular Trx. As a result, the recruitment of Trx at the specific chromatin locus is reduced thereby leading to the down regulation of Trx target genes (59).

The third arm of epigenetic control, namely the small interfering RNA-mediated post-transcriptional gene silencing is also influenced by Hsp90 chaperone complex. It has been demonstrated that Hsp90/Hsc70 chaperone complex is required for the loading of small RNA duplexes onto the Argonaute proteins (60). Its involvement in the assembly and maintenance of box C/D small nucleolar ribonucleoprotein (SnoRNP) complexes is also observed. Hsp90 along with Tah1 and Pih1 interact with Rvb1/2 to form R2TP complex, which participates in assembly of snoRNPs (61). Interestingly, while Hsp90 controls the activity of chromatin modifiers, its own activity is often regulated by non-histone methyl transferases. Such regulation provides another layer of regulation where Hsp90 is a central molecule. Recent report witnesses that SMYD2-mediated methylation of Hsp90β induces its dimerization and chaperone complex formation, which accelerates the proliferation of cancer cell (62).

Hsp90 collaborates with histone deacetylases to influence the stability of oncogenic TFs and tumor suppressors. The Hsp90–HDAC6 complex is critical for the stability of mutant p53 (63). Recent reports establish that the regulation of tumor suppressor TAp73 stability is mediated by Hsp90–HDAC1 combo protein complex. HDAC1 knockdown induces hyperacetylation of Hsp90, which disrupts the interaction between TAp73 and Hsp90 and promotes proteasomal degradation of TAp73 (64). Thus, Hsp90 influences the activity of several epigenetic modifiers as well as the micro-RNAs. Independent studies have revealed the link between cancer progression and the improper functioning of such epigenetic writers, speculating a general role of Hsp90 in cancer progression through the modulation of chromatin dynamics. However, any such direct connection between Hsp90, chromatin modification, and clinical progression of cancer is yet to be established.

## The Function of Hsp90 at Promoter Proximal Regions

The transcription machinery including RNA polymerase, transcription activators, and other factors need to be recruited at the promoter adjacent region at the onset of transcription and once transcription is over they must be dislodged from the DNA. Hsp90 actively participates at all the above steps of transcription. Genome wide ChIP-seq analysis reveals that Hsp90 is recruited at the transcription start site (TSS) of about one-third of *Drosophila* genome suggesting a general role of Hsp90 in transcription initiation (65). Interestingly, Hsp90 targeted promoters include TFs like c-myc, p53; genes involved in stress response and developmental signaling such as WNT, JNK, etc.; as well as several environmental responsive genes like Hsp70, Hsp68, and Hsp22. It is observed that Hsp90 together with negative elongation factor (NELF) represses the expression of its target genes by forming stalled RNA polymerase II at the target locus. Hsp90 inhibitory condition causes robust up-regulation of Hsp90 target genes by converting stalled RNA polymerase to the elongated form. However, Hsp90 may not have a general role in transcription as it is evident from another study where Hsp90 and Trx are co-localized only at the TSS of the actively transcribed region *Abd-B* in *Drosophila* SF4 cells (59) (Figure 1, 5) but neither it is found to be associated with Trx at the TSS of silent genes (*Dfd* or *Ubx*) nor at the TSS of house-keeping genes. There are reports, which show that Hsp90 also enhances transcriptional activation in cancer cells by binding to the DNA–protein complex. It is observed that Hsp90 interacts strongly to the hTERT promoters in telomerase positive oral cancer cell lines compared to the normal human oral keratinocytes (NHOKs) cell lines and thereby causes enhanced promoter activity of telomerase gene in cancer cells (66). Hsp90 inhibition by GA specifically destabilizes the interaction between Hsp90 and hTERT promoter causing loss of hTERT mRNA expression.

It turns out that the role of Hsp90 in transcriptional regulation begins much earlier than the recruitment of TFs or RNA pol II. It is observed that Hsp90 is involved in the steps prior to the transcription initiation, which involves precise removal of nucleosomes (Figure 1, 3). The transcriptional induction of *GAL1* is found to be delayed in Δ*hsc82* strain background due to the retention of nucleosomes at the *GAL1* promoter (67). However, the precise mechanism of how Hsp90 aids in the eviction of nucleosomes is not clear.

Consistent with the function of Hsp90 in the removal of histone proteins from several promoters, Hsp90 also removes other proteins from the promoter proximal regions. Hsp90 controls the exit of steroid hormone receptors from nuclear locus. Hsp90 and its co-chaperone p23 also play pivotal roles during the dislodging of steroid hormone receptor complexes from hormone response elements (HRE) in a hormone-dependent manner (68). First, over-expression of p23 causes significant (35-fold) reduction of GR activity *in vitro*. Similarly, Hsp90 over-expression results in modest (twofold) reduction. Second, ChIP assay shows increased recruitment of Hsp90, p23, and gluococorticoid receptor at GRE upon addition of dexamethasone. Finally, forced localization of Hsp90/p23 to HRE precludes GR-induced transcriptional activation.

In summary, Hsp90 has multifaceted cellular functions in transcription regulations. It could evict nucleosomes from the promoter and thereby makes space for loading of RNA pol II and other TFs; it could alter the heterochromatin to euchromatin states by modulating chromatin modifiers; it could give functional maturation to the TFs and regulate their nuclear entry; and finally, it could remove the TFs from the promoter proximal regions upon the completion of transcription.

## Future Perspective

In the light of the recent findings, it is becoming clear that besides the well known chaperone function Hsp90 plays significant roles at many stages of transcriptional control. However, it is not clear whether Hsp90 has a generalized role during transcription or its involvement is confined to certain specific promoters. In the later case, it would be extremely important to decipher the molecular mechanism behind such promoter specificity. It will also be interesting to unravel whether human Hsp90 also targets promoters of tumor suppressors/oncogenes. The interplay between Hsp90 and chromatin modifiers during carcinogenesis needs to be investigated. Studies focusing on whether and how human Hsp90 modulates post-transcriptional gene regulation via non-coding micro-RNAs in cancer cells demand special attention. The classical chaperone function of cytosolic Hsp90 and several newly emerged moonlighting functions of Hsp90 at the nucleus prompt us to propose that the nuclear Hsp90 could be structurally different (due to certain post-translational modification: PTM) from the cytosolic form. Identification of different PTM of Hsp90 might give us valuable handle in separating the cytosolic versus the nuclear functions of Hsp90. This field is still at its infancy and more experimentations are needed to understand the yet to be discovered newer nuclear functions of Hsp90.

## Conflict of Interest Statement

The authors declare that the research was conducted in the absence of any commercial or financial relationships that could be construed as a potential conflict of interest.
